# Nodular Swelling on the Lower Lip; Pyogenic Granuloma

**Published:** 2014-11-18

**Authors:** Abdulrasheed Ibrahim, Malachy E. Asuku

**Affiliations:** Division of Plastic and Reconstructive Surgery, Department of Surgery, Ahmadu Bello University Teaching Hospital, Zaria, Nigeria

**Keywords:** pyogenic granuloma, lower lip, surgery, cosmesis, female

## DESCRIPTION

**Figure 1:**
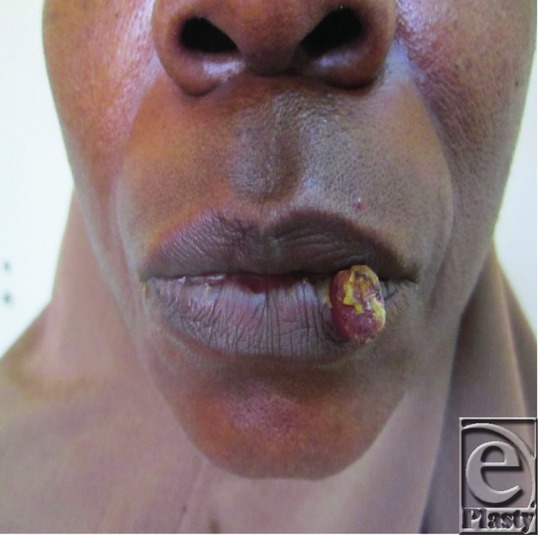
Preoperative photograph.

A 43-year-old female patient presented with a swelling of 6-weeks duration on the lower lip (Fig 1). The swelling had grown progressively, and it was associated with bleeding while eating. She was concerned about the cosmetic disfigurement and requested for a removal of the lesion.

## QUESTIONS

1. **What is the etiology of pyogenic granuloma?**
2. **How does pyogenic granuloma present?**
3. **What are the treatment options for this patient?**
4. **What are the differential diagnoses?**

## DISCUSSION

Pyogenic granuloma has been classified as a disorder of angiogenesis, although no single unifying hypothesis adequately explains the etiopathogenesis.^1^^,^^2^ It is characterized by an exuberant vascular response to an angiogenic stimulus with an increase in angiogenic growth factors such as vascular endothelial growth factor and basic fibroblast growth factor.^2^^,^^3^ It is postulated that chronic trauma (in the mouth or lip from chewing) and hormonal influences (pregnancy and oral contraceptives in oral mucosa lesions) play a significant role in the soft tissue response to vascular proliferation.^1^^,^^3^^-^5 Its frequent location in the head and neck has been attributed to the excellent blood supply of this region.^2^^,^^3^

Pyogenic granuloma is relatively common with a worldwide distribution. A higher frequency is however observed in the second and third decade of life, with peak prevalence in women.^1^^,^^3^ It accounts for 10% of all biopsy findings from the lips and oral cavity.^2^ The commonest locations are the lips, tongue, buccal mucosa, gingiva, and palate.^2^^,^^4^ Extra-oral sites have also been described; the skin of the upper and lower extremities, scalp, face, mucous membrane of the nose, eyelids, and genitalia.^6^ The classical clinical presentation is a small, deep red to beefy red nodular swelling, which is either pedunculated or sessile. It may manifest with a startling rapid growth.^5^^,^^7^ It is usually painless and soft in consistency, although older lesions tend to be firm and fibrotic. The surface may be smooth or occasionally ulcerated with a tendency for hemorrhage either spontaneously or upon slight trauma. Bleeding may be episodic, profuse, and refractory to pressure, particularly when on the lip and this, together with cosmetic factors, is a common indication for referral.^3^^,^^4^^,^^7^

**Figure 2:**
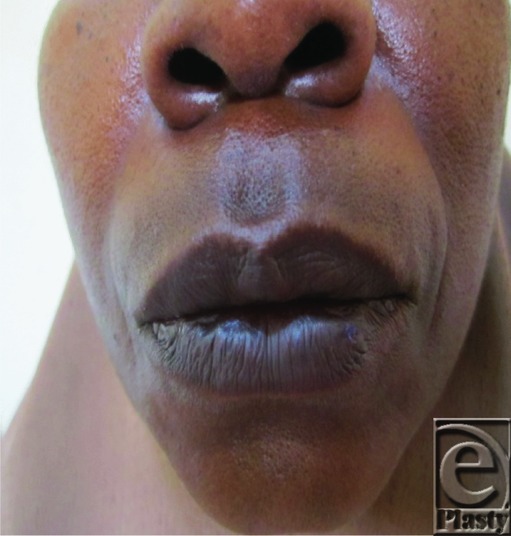
Postoperative photograph.

Management has 2 goals: diminution of the risk of recurrence and an improved cosmetic appearance. Several detailed reviews have demonstrated the application of the full spectrum of treatment options of which the standard is thought to be excision and primary closure.^3^ Advantages of surgery include a single treatment session and the lesion in its entirety is sent for pathologic examination.^8^ The patient described in this case had a complete elliptical excision and meticulous closure with 5/0 vicryl suture. The result of surgical excision is esthetically pleasing to her (Fig 2). She was followed up for 1 year after excision and no recurrence was noted. Nonsurgical treatment includes cryotherapy and laser. Cryotherapy in the form of either a liquid nitrogen spray or a cryoprobe is safe and easy technique, which is suitable for the setting of a plastic surgery out patient's clinic.^1^ Lasers have also proved to be a successful alternative. Carbon dioxide laser and flash lamp pulsed dye laser have all been used for the treatment of pyogenic granuloma with the advantages of minimal invasiveness and pain. In addition, it obviates the need of suturing.^1^ Other treatment modalities include chemical or electric cauterization and microembolization.^8^ An acceptable outcome with sclerotherapy using ethanolamine oleate has also been reported.^5^

Although pyogenic granuloma has distinct clinical features, the clinical diagnosis may often be difficult, due to lesions that appear similar.^5^ Benign lesions that should be considered in the differential diagnosis are seborrheic keratosis, furuncle, ecthyma contagiosum, and verruca vulgaris. Malignant lesions also deserve due consideration. These include squamous cell carcinoma, basal cell carcinoma, keratoacanthoma, amelanotic melanoma, and Kaposi's sarcoma.^1^^,^^3^^,^^7^

Pyogenic granulomas are benign vascular proliferations commonly seen in young female adults. An exuberant vascular response to minor trauma and hormonal influences has been implicated. Acceptable esthetic result can be achieved with surgical excision, which, if adequate, should not lead to recurrence.
